# Clinical results after hybrid coronary revascularization with totally endoscopic coronary surgery

**DOI:** 10.1186/s13019-022-01840-8

**Published:** 2022-05-03

**Authors:** Jade Claessens, Alaaddin Yilmaz, Camille Awouters, Hanne Oosterbos, Stef Thonnisen, Edouard Benit, Abdullah Kaya, Yoann Bataille

**Affiliations:** 1 Department of Cardiothoracic Surgery, Jessa Hospital, Stadsomvaart 11, Hasselt, Belgium; 2 Faculty of Medicine and Life Sciences, LCRC, UHasselt - Hasselt University, Martelarenlaan 45, Hasselt, Belgium; 3 Department of Cardiology, Jessa Hospital, Stadsomvaart 11, Hasselt, Belgium

**Keywords:** Coronary artery disease, Coronary artery bypass grafting, Percutaneous coronary intervention, Hybrid coronary revascularization

## Abstract

**Background:**

The optimal revascularization strategy remains uncertain in multivessel coronary artery disease (MVCAD). The durability of the surgical grafts should be weighed against the decreased invasiveness of percutaneous coronary intervention (PCI). Hybrid coronary revascularization (HCR), a combination of PCI and surgery, could be a feasible alternative. This study aimed to investigate the occurrence of major adverse cardiac and cerebrovascular events (MACCE) and all-cause mortality after both endoscopic coronary artery bypass grafting (Endo-CABG) and the HCR procedure.

**Methods:**

In this single-center retrospective observational study, 347 consecutive patients have been subjected to an Endo-CABG procedure, of which 103 underwent HCR between January 2016 and January 2018. A propensity score matching analysis was performed to match 103 Endo-CABG alone patients to the 103 HCR patients. The Endo-CABG procedure was performed through 3 endoscopic ports (5 mm) in the 2nd, 3rd, and 4th intercostal space and a utility port of 3 cm.

**Results:**

In both the HCR and matched endo-CABG alone group, the 30-day mortality was acceptable (0% in the HCR group and 1.94% in the matched Endo-CABG alone group, *p* = 0.155). Additionally, the occurrence of MACCE after a mean follow-up of 1188 ± 538 days was similar in both groups (9.71% and 11.65% for the HCR and matched Endo-CABG alone group, respectively, *p* = 0.652). Still, the long-term all-cause mortality over this period was significantly higher in the matched Endo-CABG alone group (2.91% after the HCR procedure and 11.65% after matched Endo-CABG alone, *p* = 0.002).

**Conclusion:**

HCR has some advantages over Endo-CABG alone regarding the all-cause mortality, cross-clamping time, intensive care unit, and hospital length of stay. Therefore, HCR may be a suitable alternative therapy for patients with MVCAD.

## Introduction

Coronary artery disease (CAD) has typically been treated either medically, with percutaneous coronary intervention (PCI), or with coronary artery bypass grafting (CABG). As advances in stent technology and minimally invasive surgery have developed, hybrid coronary revascularization (HCR) has emerged as an alternative treatment for multivessel coronary artery disease (MVCD) [1, 2]. Classically, it combines a minimally invasive sternal-sparing coronary artery bypass for a left internal mammary artery (LIMA) to the left anterior descending (LAD) coronary artery and PCI to non-LAD coronary arteries, mainly right coronary artery (RCA) [3]. However, the use of bilateral internal mammary arteries (BIMA), in situ or a composite Y- or T-graft, in multivessel coronary lesions in addition to LAD is also possible [4–6]. The 2014 American College of Cardiology/American Heart Association guidelines for stable ischemic heart disease state that HCR is reasonable in specific patients: patients with limitations to traditional CABG, such as a heavily calcified proximal aorta or poor target vessels for CABG; patients with a lack of suitable graft conduits; and patients with unfavorable LAD artery for PCI (i.e., excessive vessel tortuosity or chronic total occlusion) [7]. Several studies state that HCR outcomes are similar to CABG alone [1–3, 7, 8]. However, all these studies used a conventional sternotomy, a mini-sternotomy, or a thoracotomy during the surgery. Recently, Yilmaz et al. introduced a newly developed totally endoscopic CABG (Endo-CABG) using only endoscopic instruments [9]. They observed a lower 30-day mortality rate when compared to traditional CABG. This technique may offer benefits since it is less expensive and less time-consuming than robotic procedures [9]. Therefore, the purpose of this retrospective study is to report the initial experience of short- and long-term clinical outcomes in a cohort of patients undergoing HCR with the newly developed Endo-CABG procedure as surgical treatment. We hypothesize that major adverse cardiac and cerebrovascular events (MACCE) and mortality rates are lower after HCR compared to Endo-CABG alone.

## Methods

In this single-center observational study, the clinical outcomes after HCR and Endo-CABG alone were retrospectively investigated. The protocol conforms to the ethical guidelines imposed by the 1975 Declaration of Helsinki. The study was approved by the ethics committee of the Jessa hospital and Hasselt university on February 8, 2021 (2020/188).

**Fig. 1:**
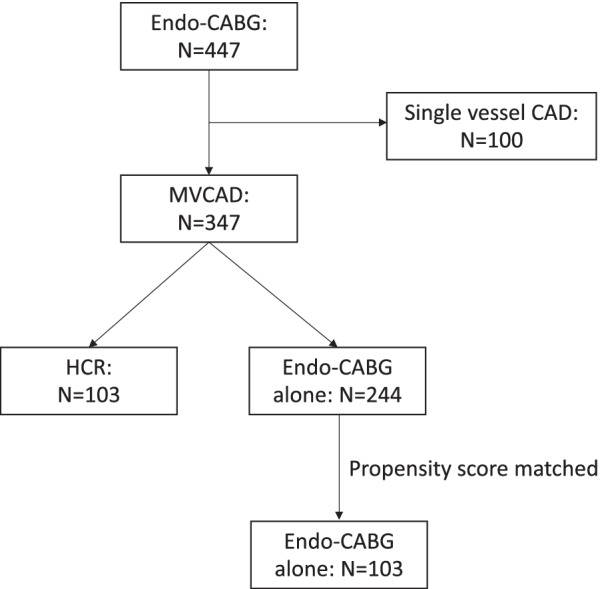
Flowchart. From January 2016 until January 2018, 447 patients underwent an endoscopic coronary artery bypass graft (Endo-CABG) procedure. Of these patients, 366 had multivessel coronary disease (MVCAD). Hybrid coronary revascularization (HCR) was performed in 103 MVCAD patients while 263 underwent an Endo-CABG alone

All 347 consecutive patients with MVCD who underwent coronary surgery in Jessa Hospital from January 2016 to January 2018 were enrolled. Altogether, 244 patients have been subjected to Endo-CABG alone and 103 to HCR (Fig. 1). The definition of MVCD is any stenosis of > 70% of the coronary lumen in more than one artery or left main stenosis of ≥ 50% [10]. The lesions were assessed through angio-visual assessment. The decision to proceed with HCR or Endo-CABG alone was made only after discussion between the heart team and the patient. Non-elective cases (acute coronary syndrome or acute myocardial infarction) were excluded. At the Jessa hospital, no CABG procedure by sternotomy is performed. All patients were completely revascularized after Endo-CABG or HCR. The data were gathered from the patients' medical files and entered into a Castor EDC database [11]. This database includes patient characteristics and history, indications for the procedure, pre-, intra-, and postoperative variables, follow-up variables, and PCI and Endo-CABG procedure data.

The Endo-CABG operative technique was previously described in detail [9]. Moreover, a Y-graft construction can be created by performing an end to side anastomosis of the free RIMA to the in-situ LIMA. This procedure is performed on-pump without robotic assistance. The PCI procedure was not guided by imaging or physiology according to standard technique via radial access [12].

Patients suffering from MVCD will undergo HCR when lesions exceed available arterial grafts. In that case, PCI is preferred over saphenous grafts since the longevity of the saphenous grafts is relatively poor [13–15].

Standard HCR is applied when the PCI is performed after the surgery. When PCI is performed before the surgery, it is referred to as reverse HCR. The order and timing for the Endo-CABG and PCI were determined by the patient's coronary anatomy and are joint decisions between the surgeon and interventional cardiologist. However, patients with critical non-LAD stenosis will typically undergo reverse HCR. In addition, most patients will undergo the procedures during two different hospitalizations for logistic reasons. For patients undergoing reverse HCR, Endo-CABG was performed without interruption of dual antiplatelet therapy to avoid stent thrombosis. Dual antiplatelet therapy included Asaflow (80 mg) and Clopidogrel (75 mg) once daily. When PCI was performed after the Endo-CABG, 600 mg Clopidogrel was administered at hospital admission.

The primary endpoints are the occurrence of MACCE at 30 days and the long-term all-cause mortality. MACCE is a composite of cardiac death, myocardial infarction, stroke, and revascularization. Cardiac death is defined as death after myocardial infarction, sudden cardiac death, heart failure, stroke, cardiac hemorrhage, and death due to cardiac procedures [16]. Secondary endpoints are peri- and postoperative bleeding, hospital and intensive care unit length of stay (LOS), graft patency, and postoperative complications.

Statistical analyses were performed using R Core Team (2021) R: A language and environment for statistical computing. R Foundation for Statistical Computing, Vienna, Austria. Statistical significance is defined as a p-value of < 0.05. Data normality was evaluated using QQ-plots and Shapiro–Wilk tests. All data were represented as mean ± standard deviation (SD), median (interquartile range (IQR)) or numbers (n), and percentages (%) as appropriate. Differences between groups were compared using a chi-square test, Mann–Whitney U test, Wilcoxon Signed-Rank test, and t-tests also as appropriate. A Kaplan–Meier analysis was performed to estimate the probability of survival and occurrence of MACCE past given time points. A propensity score matching was implemented using nearest pairing to correct for baseline differences between the two groups.

## Results

In total, 347 consecutive patients were enrolled in the analysis, of which 244 (70.32%) were in the Endo-CABG alone, and 103 (29.68%) were in the HCR group. The mean follow-up time was 1188 ± 538 days. Of the patients undergoing HCR, 44 (42.27%) received PCI first (reverse HCR) and 59 (57.28%) CABG first (standard HCR). The mean age of the included patients was 67.62 ± 10.08 and 67.67 ± 10.15 years for respectively the Endo-CABG alone and the HCR group (*p* = 0.04), and 85.01% of all enrolled patients were male. The mean European System for Cardiac Operative Risk Evaluation (Euroscore) II was 2.24 ± 2.60 in the HCR group and 2.78 ± 2.97 in the Endo-CABG alone group. This Euroscore II corresponds to a moderate operative risk in both groups. A significant difference was found in baseline characteristics between HCR and Endo-CABG alone patients for age, preoperative PCI, chronic kidney disease, and left main disease (*p* = 0.040, *p* = 0.020, *p* = 0.034, and *p* < 0.001, respectively). After propensity score matching, there were no differences in baseline characteristics. All demographic variables are represented in Table 1.

**Table 1:** Baseline characteristics

		Unmatched patients		Matched patients	
	HCR (N = 103)	Endo-CABG (N = 244)	*p* value	Endo-CABG (N = 103)	*p* value
Age (years)	65.23 ± 9.96	67.67 ± 10.15	**0.040**	65.72 ± 9.61	0.722
Octogenarians	8 (7.77)	34 (13.93)	0.107	7 (6.80)	0.789
Gender (male)	88 (85.44)	207 (84.84)	0.886	89 (86.41)	0.841
BMI (kg/m^2^)	27.94 ± 4.18	27.74 ± 4.31	0.680	28.10 ± 4.08	0.781
EuroSCORE II	2.24 ± 2.60	2.78 ± 2.97	0.243	2.41 ± 3.02	0.895
*NYHA*			0.192		0.225
I	62 (60.19)	123 (50.41)		50 (48.54)	
II	35 (33.98)	94 (38.52)		43 (41.75)	
III	4 (3.88)	24 (9.84)		9 (8.74)	
IV	2 (1.94)	2 (0.82)		1 (0.97)	
LVEF (%)	56.24 ± 9.62	55.81 ± 11.71	0.782	56.84 ± 13.09	0.770
*Comorbidities*					
Smoking	22 (21.36)	69 (28.28)	0.327	29 (28.16)	0.520
DM type I	0	2 (0.82)	0.628	0	0.656
DM type II	32 (31.07)	79 (32.38)		35 (33.98)	
Atrial hypertension	66 (64.07)	159 (65.16)	0.847	68 (66.02)	0.770
Dyslipidemia	89 (86.41)	200 (81.97)	0.311	85 (82.52)	0.442
Family cardiac history	52 (50.49)	120 (49.18)	0.664	58 (56.31)	0.552
Extracardiac arteriopathy	15 (14.56)	44 (18.03)	0.432	19 (18.45)	0.453
Atrial fibrillation	9 (8.74)	25 (10.25)	0.666	11 (10.68)	0.638
Pacemaker	1 (0.97)	8 (3.29)	0.193	3 (2.91)	0.360
Chronic kidney disease	11 (10.68)	49 (20.08)	**0.034**	14 (13.59)	0.522
Previous PCI	18 (17.48)	72 (29.51)	**0.020**	18 (17.48)	0.856
Previous cardiac surgery	2 (1.94)	3 (1.23)	0.611	1 (0.97)	0.561
*Neurological history*			0.360		0.241
CVA	2 (1.94)	16 (6.56)		7 (6.80)	
TIA	3 (2.91)	6 (2.46)		1 (0.97)	
Left main disease	18 (17.48)	87 (35.66)	** < 0.001**	23 (13.59)	0.383

Data are presented as mean ± SD or as number (%)

BMI: body mass index; COPD: chronic obstructive pulmonary disease; CVA: cerebrovascular accident; DM: diabetes mellitus; Endo-CABG: endoscopic coronary artery bypass graft; EuroSCORE II: European System for Cardiac Operative Risk Evaluation; NYHA: New York Heart Association functional classification; LVEF: left ventricular ejection fraction; PCI: percutaneous coronary intervention; TIA: transient ischemic attack

In the Endo-CABG alone group, a partial or complete sternotomy conversion occurred in 1.30% and 0.82%, respectively, while in the HCR group, in 1.94% and 0.97%, respectively. Perioperative data are represented in Table 2.

**Table 2:** Outcomes of the HCR and Endo-CABG alone groups

		Unmatched patients		Matched patients	
	HCR (N = 103)	Endo-CABG (N = 244)	*p* value	Endo-CABG (N = 103)	*p* value
*General*					
30-day mortality	0	5 (2.05)	0.083	2 (1.94)	0.155
All-cause mortality	3 (2.91)	35 (14.34)	**0.002**	12 (11.65)	**0.016**
30-day MACCE	2 (1.94)	11 (4.5)	0.250	3 (2.91)	0.651
Cardiac death	0	5 (2.05)		2 (1.94)	
Myocardial infarction	0	2 (0.82)		1 (0.97)	
Stroke	1 (0.97)	2 (0.82)		0	
Revascularization	1 (0.97)	1 (0.38)		0	
Long-term MACCE	10 (9.71)	34 (13.93)	0.280	12 (11.65)	0.652
Cardiac death	1 (0.97)	11 (4.51)		4 (3.88)	
Myocardial infarction	1 (0.97)	8 (3.28)		2 (1.94)	
Stroke	1 (0.97)	8 (3.28)		3 (2.91)	
Revascularization	7 (6.80)	7 (2.87)		3 (2.91)	
Readmissions	24 (23.30)	54 (22.13)	0.898	27 (26.21)	0.873
Arrhythmia	6 (5.83)	12 (4.92)			
AF	3 (2.91)	10 (4.10)			
VES	1 (0.97)	1 (0.38)			
Tachycardia	2 (1.94)	1 (0.38)			
Heart failure	3 (2.91)	9 (3.69)			
STEMI	0	2 (0.82)			
NSTEMI	2 (1.94)	4 (1.64)			
Pericarditis	1 (0.97)	3 (0.82)			
Pleuritis	0	4 (1.64)			
Syncope	0	2 (0.82)			
Stable angina	7 (6.80)	12 (4.92)			
PCI	6 (5.83)	6 (2.46)			
No intervention	1 (0.97)	5 (2.46)			
Unstable angina	1 (0.97)	1 (0.38)			
Aspecific complaints	3 (2.91)	6 (2.46)			
*Endo-CABG*					
Number of bypasses	2.39 ± 0.88	2.96 ± 0.85	** < 0.001**	2.88 ± 0.78	** < 0.001**
Graft			–		–
LIMA	58 (56.31)	–		–	
RIMA	3 (2.91)	–		–	
BIMA in situ	9 (8.74)	93 (38.11)		42 (40.78)	
Y-graft	33 (32.04)	151 (61.88)		61 (59.22)	
ICU LOS (h)	70.62 ± 46.12	91.44 ± 101.41	**0.009**	82.62 ± 61.02	0.115
Ventilation time (h)	10.43 ± 23.03	17.53 ± 44.79	0.054	16.38 ± 39.46	0.192
Hospital LOS (days)	7.62 ± 3.74	9.46 ± 8.76	**0.008**	8.27 ± 3.92	0.222
Cross-clamping time (min)	50.55 ± 25.61	64.00 ± 22.33	** < 0.001**	62.32 ± 21.49	** < 0.001**
CPB time (min)	92.87 ± 38.81	110.40 ± 32.78	** < 0.001**	109.4 ± 33.7	**0.001**
Perioperative bleeding (ml)	445.10 ± 442.81	474.60 ± 513.66	0.593	531.1 ± 641.46	0.270
Bleeding 24 h (ml)	541.00 ± 724.84	651.50 ± 680.57	0.191	645.0 ± 721.49	0.306
LVEF					
> 50%	54 (52.43)	85 (35.12)		36 (36.28)	
31–50%	19 (18.45)	34 (14.05)		10 (10.78)	
21–30%	1 (0.97)	4 (1.65)		2 (1.96)	
< 20%	0	1 (0.41)		1 (0.98)	
No TTE	29 (28.16)	118 (48.76)		51 (50.00)	
New-onset AF	24 (23.30)	72 (29.51)	0.238	28 (27.18)	0.521
Electric cardioversion	22 (21.36)	36 (14.75)	0.132	18 (17.48)	0.481
In hospital neurological complications			0.524		0.844
CVA	1 (0.97)	2 (0.82)		1 (0.97)	
TIA	0	1 (0.38)		0	
Early revision	6 (5.83)	21 (8.61)	0.377	11 (10.78)	0.206
Late revision	2 (1.94)	1 (0.41)	0.159	1 (0.97)	0.561
Graft failure	6 (5.83)	8 (3.28)	0.271	3 (2.91)	0.307
*PCI*					
Number of stents	1.42 ± 0.75	–	–	–	–
Neurological complications					
CVA	0	–	–	–	–
TIA	0	–	–	–	–
In-stent stenosis	3 (2.91)	–	–	–	–
Hospital LOS	1.96 ± 2.28	–	–	–	–

Data are presented as mean ± SD or as number (%)

AF: atrial fibrillation; BIMA: bilateral internal mammary artery; CPB: cardiopulmonary bypass; CVA: cerebrovascular accident; Endo-CABG: endoscopic coronary artery bypass graft; HCR: hybrid coronary revascularization; ICU: intensive care unit; LOS: length of stay; LIMA: Left internal mammary artery; LVEF: left ventricular ejection fraction; MACCE: major adverse cardiac and cerebrovascular events; NSTEMI: non-ST-elevated myocardial infarction; PCI: percutaneous coronary intervention; RIMA: Right internal mammary artery; STEMI: ST-elevated myocardial infarction; TIA: transient ischemic attack; TTE: transthoracic echocardiogram; VES: ventricular extrasystole

**Fig. 2:**
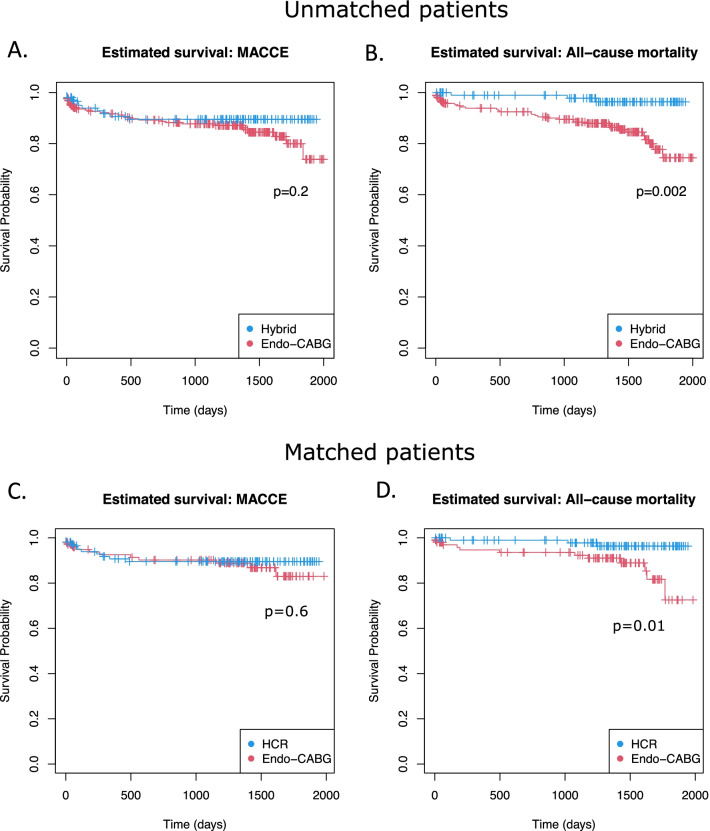
Estimated survival (Kaplan–Meier) of major adverse cardiac and cerebrovascular events (MACCE) and all-cause mortality in unmatched (**a, b**) and propensity score matched patients (**c, d**). Unmatched endoscopic coronary artery bypass grafting (Endo-CABG): n = 244; matched Endo-CABG: n = 103; Hybrid: n = 103. A *p* value of < 0.05 is considered statistically significant

The occurrence of MACCE after 30 days and a follow-up of 1188 ± 538 days was not significantly different between HCR and matched Endo-CABG alone (30 days: respectively 2 (1.94%) versus 3 (2.91%) for HCR and matched Endo-CABG patients; *p* = 0.651; long term: respectively 10 (9.71%) versus 12 (11.65%) for HCR and matched Endo-CABG patients; *p* = 0.652). The freedom of MACCE at the end of follow-up is 83.0% after matched Endo-CABG alone and 89.5% after HCR (*p* = 0.6, Fig. 2c). Four (3.88%) patients suffered cardiac death in the matched Endo-CABG group, and two (1.94%) patients endured a myocardial infarction. The occurrence of stroke was 2.91%, and revascularization was needed in three (2.87%) patients. There was one (0.97%) cardiac death in the HCR group, one myocardial infarction (0.97%), and one stroke (0.97%). In the HCR group, there was a need for revascularization in seven (6.80%) patients. On the other hand, with 12 (11.65%) deaths in the matched Endo-CABG alone group and three (2.91%) deaths in the HCR group, the long-term all-cause mortality is significantly lower in the group of patients that received HCR compared to those that underwent Endo-CABG alone (*p* = 0.016). The estimated overall survival at the end of follow-up is 72.5% after Endo-CABG and 96.3% after HCR (*p* = 0.01, Fig. 2d). Additionally, 31.3% of the deaths in the matched Endo-CABG group were octogenarians. The proportion of octogenarians for the all-cause mortality in the hybrid group was 66.6%. Moreover, of all octogenarians in the matched Endo-CABG group, there was a mortality rate of 31.3%. On the other hand, in the HCR group, the all-cause mortality rate of octogenarians was 25%.

In addition to the four patients that suffered cardiac death, the cause of death was not defined in two patients. Moreover, a stroke caused the death of two patients, and another one died due to postoperative bleeding after carotid endarterectomy. Additionally, one patient died due to coronavirus disease 2019 (COVID-19) and one due to lung disease. One patient of the HCR group passed away due to multiorgan failure and another from a myocardial infarction. The last patient of the HCR group died of an unknown cause. All outcomes are represented in Table 2.

The intensive care unit LOS, ventilation time, and hospital LOS were not significantly different between the matched Endo-CABG and HCR groups. Moreover, the study groups did not differ between postoperative parameters among new-onset atrial fibrillation, neurological dysfunction, and perioperative and 24 h bleeding. Blood chemistry and hematology data are shown in Table 3. Finally, no significant difference in revisions or graft failure was found between the aforementioned groups. In the HCR group, no patients died within 30 days after the Endo-CABG or PCI procedure. The mean number of stents placed is 1.42 ± 0.75. Three patients got in-stent stenosis. No one developed neurological problems after their stent placement (Table 2).

**Table 3:** Blood chemistry and hematology data

		Unmatched patients		Matched patients	
	HCR (N = 103)	Endo-CABG (N = 244)	*p* value	Endo-CABG (N = 103)	*p* value
*Hemoglobin (g/dl)*					
Preoperative	13.97 ± 1.45	13.58 ± 1.72	**0.031**	13.97 ± 1.60	0.995
Post-operative	11.03 ± 1.03	10.60 ± 1.25	**0.001**	10.69 ± 1.22	**0.036**
p-value	** < 0.001**	** < 0.001**		** < 0.001**	
*Creatinine (mg/dl)*					
Pre-operative	1.07 ± 0.29	1.24 ± 0.88	**0.007**	1.23 ± 1.02	0.1134
Post-operative	0.99 ± 0.29	1.17 ± 0.87	**0.008**	1.16 ± 1.06	0.1485
p-value	**0.002**	0.088		**0.034**	

Data are presented as mean ± SD

Endo-CABG: endoscopic coronary artery bypass graft; HCR: hybrid coronary revascularization

For patients of the HCR group who underwent reverse HCR (N = 44), the mean number of days between the two interventions was 46.23 ± 39.48 days. For those who received standard HCR (n = 59), the mean number of days in-between was 59.07 ± 57.42 days (*p* < 0.001). The CPB time was significantly shorter in the reverse HCR group (*p* = 0.034). Additionally, there was a significant difference in graft failure. It occurred in 10.17% after standard HCR while no graft failure was seen in the reverse HCR group (*p* = 0.029). However, there was no significant difference in peri- and postoperative bleeding between standard and reverse HCR (Perioperative: *p* = 0.266; Postoperative: *p* = 0.812). All outcomes of standard and reverse HCR are represented in Table 4.

**Table 4:** Outcomes in reverse and standard HCR

	Reverse HCR (N = 44)	Standard HCR (N = 59)	*p* value
*General*			
Days between procedures	46.23 ± 39.48	59.07 ± 57.42	**< 0.001**
30-day mortality	0	0	
All-cause mortality	0	3 (1.75)	0.129
30-day MACCE	1 (2.27)	1 (1.70)	0.834
Cardiac death	0	0	
Myocardial infarction	0	0	
Stroke	1 (2.27)	0	
Revascularization	0	1 (1.70)	
Long-term MACCE	3 (6.82)	7 (11.86)	0.392
Cardiac death	0	0	
Myocardial infarction	1 (2.27)	1 (1.70)	
Stroke	1 (2.27)	0	
Revascularization	1 (2.27)	6 (10.17)	
*Endo-CABG*			
ICU LOS (h)	72.70 ± 44.84	69.1 ± 47.35	0.697
Ventilation time (h)	8.09 ± 7.93	11.75 ± 29.56	0.442
Hospital LOS (days)	8.19 ± 4.03	7.27 ± 3.51	0.286
Cross-clamping time (min)	50.10 ± 26.03	50.88 ± 25.54	0.885
CPB time (min)	83.26 ± 37.29	99.71± 38.72	**0.034**
Perioperative bleeding (ml)	506.30 ± 550.08	399.70 ± 340.76	0.266
Bleeding 24h (ml)	560.30 ± 604.72	527.00 ± 805.99	0.812
LVEF			
> 50%	16 (36.36)	38	64.41)
31–50%	12 (27.27)	7	(11.86)
21–30%	0	1 (1.69)	
< 20%	0	0	
No TTE	16 (36.36)	13 (22.03)	
New-onset AF	10 (22.72)	14 (23.73)	0.956
Electric cardioversion	10 (22.72)	12 (20.34)	0.770
In hospital neurological complications			0.352
CVA	1 (2.27)	0	
TIA	0	0	
Early revision	4 (9.09)	2 (3.39)	0.222
Late revision	1 (2.27)	1 (1.70)	0.834
Graft failure	0	6 (10.17)	**0.029**
*PCI*			
Number of stents	1.51 ± 0.83	1.36 ± 0.69	0.318
In-stent stenosis	2 (4.55)	1 (1.70)	0.678
Hospital LOS	2.53 ± 2.95	1.58 ± 1.59	0.069

Data are presented as mean ± SD or as number (%)

AF: atrial fibrillation; CPB: cardiopulmonary bypass; CVA: cerebrovascular accident; Endo-CABG: endoscopic coronary artery bypass graft; HCR: hybrid coronary revascularization; ICU: intensive care unit; LOS: length of stay; LVEF: left ventricular ejection fraction; MACCE: major adverse cardiac and cerebrovascular events; PCI: percutaneous coronary intervention; TIA: transient ischemic attack; TTE: transthoracic echocardiogram

## Discussion

In this study comparing HCR to Endo-CABG, we found that the occurrence of MACCE after 30-days and a follow-up of 1186 ± 538 days did not significantly differ between HCR and matched Endo-CABG alone groups. Secondly, the all-cause mortality was significantly higher in the matched Endo-CABG alone group. Additionally, in reverse HCR, the CPB time was significantly shorter, and the peri- and postoperative bleeding did not differ compared to standard HCR. Lastly, graft failure was detected to a greater extent after standard HCR.

The occurrence of MACCE did not significantly differ between HCR and matched Endo-CABG alone patients after 30 days and a long-term follow-up of 1186 ± 538 days. After 30 days, the occurrence of MACCE was 1.94% and 2.91% for HCR and matched Endo-CABG alone, respectively. A recent meta-analysis (n = 2245) showed that in patients with multivessel coronary disease, the risk of MACCE after HCR and CABG was similar between the two groups (respectively 3.6% and 5.4%, 95% CI 0.24–1.16) [1]. On the other hand, our study showed a significantly lower long-term all-cause mortality in patients who underwent HCR than those who received bypass surgery without PCI. This result contradicts two meta-analyses that found no difference in mortality after a maximum of one-year follow-up [1, 8]. The all-cause mortality in octogenarians in the Endo-CABG group (31.3%) was higher than the 25% reported by Sen et al*.* after a follow-up of 4 years. However, this 25% mortality in octogenarians was similar to the HCR group [17].

In the previously conducted trials, HCR patients had a shorter hospital LOS [1, 8] which was not the case in our study. When comparing the hospital LOS (seven days for HCR and eight days for Endo-CABG alone) with the median hospital LOS after conventional CABG in Belgium (11 days), there is a reduction in our study [18]. Another study with conventional CABG reports a similar hospital LOS [19] while it is longer in this study than after robotic CABG (5 days) [20]. Additionally, the ICU LOS was relatively long compared to other studies investigating minimally invasive procedures [21–23]. In general, LOS is a good indicator of hospital efficacy, but a longer LOS can also be explained by disparities in government payment systems. An increase in bed occupancy is financially favorable in the Belgian healthcare system. In addition, because our institute lacks a distinct intermediate care unit (MCU), ICU LOS reflects the total time spent in both the ICU and the MCU [24].

Our baseline characteristics resemble the ones found in previous studies [1, 8]. Other postoperative complications in the previous studies, such as postoperative blood loss, new-onset AF, neurological dysfunction, ventilation time, and CPB time, do not significantly differ among the study groups. However, in our study, the cross-clamping time was significantly longer when Endo-CABG was performed alone, and fewer bypasses were needed in the HCR group. Additionally, the mean cross-clamping time in the HCR group was lower than in another HCR study in which only one bypass (LIMA-LAD) was created (50.55 ± 25.61 versus 57 (38–129), in our research and Bonaros et al*.,* respectively) [25]. This result indicates that even though multiple bypasses were made in the HCR group of this study, cross-clamping time was still acceptable.

The ideal sequence of PCI and CABG is an important debate. The graft patency can be checked during the PCI when standard HCR is applied, but there is a risk of lesion instability in the waiting period between surgery and PCI. In reverse HCR, the dual antiplatelet therapy is continued to avoid stent thrombosis, increasing the risk of surgical bleeding [26]. However, we did not observe a significant difference in bleeding between standard and reverse HCR.

When further comparing the clinical outcomes, we found a significantly lower CPB time in the reverse HCR group. The other postoperative results, i.e., clamping and ventilation times, are not significantly different. Interestingly, the peri- and postoperative bleeding was not higher in the reverse HCR group despite the use of Clopidogrel. Prolonged CPB time has been associated with postoperative complications, such as heart failure, impaired coagulation, and renal, pulmonary, and neurological dysfunction [27]. Nevertheless, our study did not observe these complications, which could indicate that executing PCI before CABG is preferred. When Endo-CABG is performed before the PCI, the patency of the grafts is checked during the PCI procedure, and failure is discovered to a greater extent. However, in reverse HCR, there is no graft control so that graft failure could remain undetected.

Overall, the procedure sequence did not dramatically influence the clinical outcomes of the patients in this observational study. Though, it is important to mention that the preferred applied sequence of HCR depends not only on the best postoperative outcomes it would provide but also on the patient's clinical presentation, such as the location and severity of the lesions [28].

Due to the retrospective nature of this study, it is questionable whether the decision for the HCR treatment causes the observed difference in mortality. Therefore, interpretation of these survival curves needs to be done carefully. Additionally, this study is prone to a high amount of selection bias since the order and timing of the Endo-CABG and PCI were determined by the patient's coronary anatomy and are joint decisions between the surgeon and interventional cardiologist. Moreover, twice as many patients were enrolled into the Endo-CABG group compared to the HCR group. The assessment of the lesions was mainly performed visually. However, a hemodynamic evaluation would improve the decision-making. Another limitation of this study is that some patients were followed up at another hospital, so follow-up data was unavailable.

A prospective randomized controlled trial is needed to investigate the aforementioned findings further.

## Conclusion

This observational trial is the first to examine the value of on-pump totally endoscopic pan-arterial surgical revascularization without robotic assistance in a setting of HCR or coronary surgery alone. HCR with Endo-CABG seems to be a suitable alternative therapy for elective patients with CAD since it provides an excellent short-term outcome. Additionally, HCR has some advantages over Endo-CABG alone regarding the cross-clamping time, intensive care unit, and hospital LOS. However, a prospective trial should be performed to confirm these results.

## Data Availability

The datasets used and/or analysed during the current study are available from the corresponding author on reasonable request.
